# Proteostasis and Proteotoxicity in the Network Medicine Era

**DOI:** 10.3390/ijms21176405

**Published:** 2020-09-03

**Authors:** Marta Lualdi, Tiziana Alberio, Mauro Fasano

**Affiliations:** Department of Science and High Technology and Center of Bioinformatics, University of Insubria, I-21052 Busto Arsizio, Italy; marta.lualdi@uninsubria.it (M.L.); tiziana.alberio@uninsubria.it (T.A.)

**Keywords:** neurodegeneration, proteostasis, systems biology, network medicine, network pharmacology

## Abstract

Neurodegenerative proteinopathies are complex diseases that share some pathogenetic processes. One of these is the failure of the proteostasis network (PN), which includes all components involved in the synthesis, folding, and degradation of proteins, thus leading to the aberrant accumulation of toxic protein aggregates in neurons. The single components that belong to the three main modules of the PN are highly interconnected and can be considered as part of a single giant network. Several pharmacological strategies have been proposed to ameliorate neurodegeneration by targeting PN components. Nevertheless, effective disease-modifying therapies are still lacking. In this review article, after a general description of the PN and its failure in proteinopathies, we will focus on the available pharmacological tools to target proteostasis. In this context, we will discuss the main advantages of systems-based pharmacology in contrast to the classical targeted approach, by focusing on network pharmacology as a strategy to innovate rational drug design.

## 1. Introduction

Proteins are major players in the maintenance of cellular homeostasis and display an almost endless variety of functions. Protein function is tightly dependent on the ability of a protein to acquire and maintain a specific structure, which results from the folding of the polypeptide chain in a process mainly guided by its primary aminoacidic sequence [1]. However, the vast majority of proteins display a complex structure and need assistance to obtain the final correct conformation. Thus, in physiologic conditions, several proteins (i.e., the molecular chaperones) assist the folding process in order to avoid inappropriate interactions leading to misfolded states [2]. Their activity is even more crucial when facing cellular stress conditions, which favour protein misfolding and aggregation. Mistakes in protein folding can indeed result in protein loss-of-function and/or aberrant aggregation, with detrimental consequences for the whole cell homeostasis.

The continual maintenance of an active pool of functional proteins in a cellular system is called “proteostasis” [3]. Cellular proteostasis involves many pathways: (i) protein synthesis, (ii) protein folding, (iii) refolding of partially unfolded proteins, and (iv) sequestration and disposal of irreversibly unfolded/unneeded proteins. These pathways include hundreds of enzymes and specialized proteins.

Failures in the correct proteostasis dramatically affect cellular functions, leading to the development of many diseases. In this frame, post-mitotic cells, such as neurons, are particularly vulnerable to improper cellular proteostasis. The accumulation of toxic protein aggregates in the form of extra- and/or intra-neuronal inclusions, indeed, represents a pathogenetic hallmark in neurodegenerative diseases such as Alzheimer’s disease (AD) and Parkinson’s disease (PD) [4].

The proteostasis network can be therapeutically targeted in different ways in order to partially restore the correct protein homeostasis and to reduce cell death; for instance, molecular chaperone activity can be directly modulated by small molecules [5]. This targeted pharmacological approach represents one of the possible strategies to ameliorate and slow down neurodegeneration. However, the reductionist view of this classical “magic bullet” approach, in which the drug represents the key and the target is the lock, entails many limitations that became evident during the last two decades and were mirrored by a low number of newly approved drugs [6]. Opposite to this view is the network-centric approach in pharmacology, which is based on the fact that both diseases and organisms are complex systems made up of thousands of interacting components, so that one drug usually displays multiple targets [7]. In this frame, an alternative possibility is the use of a systems biology approach to identify novel candidate pathways to be further investigated and targeted. The use of untargeted “omics” discovery strategies and their integration through the tools of systems biology represent crucial concepts in the field of modern network medicine and an important step towards the application of a “personalized medicine” approach to diseases [8,9].

In this review article, after a general overview of the main components of the proteostasis network and its failure in neurodegenerative proteinopathies, we will discuss the available tools to target proteostasis and focus on the role of systems biology in unveiling novel candidate therapeutic targets.

## 2. The Proteostasis Network

It has been estimated that human ribosomes synthesize the bulk of the cellular proteome at a rate of five to six amino acids per second, producing more than a billion proteins per single human cell [10]. This process is not error-free, and approximately one in twenty newly translated proteins contain a sequence error, which can cause misfolding and/or reduced stability [11]. Aberrant protein products can also result from posttranscriptional errors (e.g., splicing) and from the production of defective mRNAs, which elude the RNA surveillance quality control pathways [12]. Moreover, the rate of protein synthesis, which can be regulated in response to a variety of stimuli (e.g., stress conditions), is also a crucial aspect, and the ribosome is emerging as a central quality control organelle by checking the conformation of the nascent protein and recruiting protein folding and translocation machineries. During protein translation, the so-called “optimal codons” are recognized by highly available tRNAs, which speeds up the translation process. By contrast, “non-optimal” codons are recognized by less abundant tRNAs, thus slowing down translation at key structural motifs in order to facilitate proper protein folding [13]. Moreover, reduced tRNAs availability may lower the translation rate, thus favouring protein aggregation [14]. The protein folding process, which is both co- and post-translational, is also crucial in determining protein fate, such as the maintenance of the correct conformational state and the disposal of unwanted protein products.

**Figure 1:**
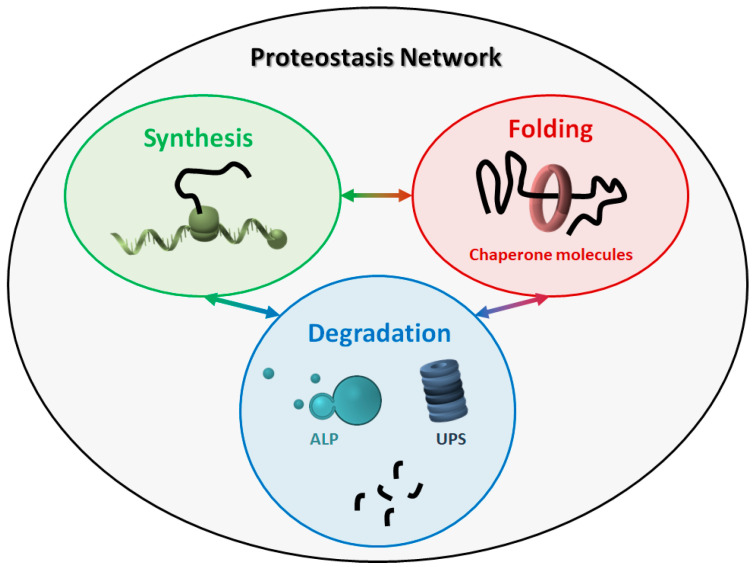
The proteostasis network (PN). The PN includes three modules, namely protein synthesis, folding, and degradation. The three modules are tightly connected to one another, and all components work together to maintain the correct protein homeostasis. ALP: autophagy-lysosome pathway. UPS: ubiquitin-proteasome system.

The proteostasis network (PN) comprises three major modules (Figure 1), which govern the three main processes involved in protein homeostasis: synthesis, folding, and degradation. Indeed, protein products must be translated from their corresponding mRNAs, reach their final conformation (linked to their function), and eventually be degraded, based on their programmed lifespan. A central mechanism, which encompasses all steps in proteostasis, is the ability of proteins to create either stable or transient interactions, thus forming specific complex structures (e.g., cytoskeleton proteins) and/or multi-protein complexes with specific activities (e.g., enzymes). Thus, proteins are physiologically prone to aggregate with one another in a controlled way, while their aberrant aggregation leads to the accumulation of toxic products.

Virtually all molecules involved in the three major modules can be considered as members of the PN. The PN components belonging to the three modules work in a coordinated way to maintain a healthy cellular proteome. Some components are generally required, such as ribosomes and the proteasome, while some others are more specific, depending on the type of proteins and/or the cellular compartment involved. For instance, some organelles (e.g., the endoplasmic reticulum and mitochondria) need specialized chaperone molecules for protein folding and trafficking [15,16]. Overall, due to this complexity, the exact composition of the human PN is difficult to define, even though an estimate of about 2000 components has been recently proposed [17].

**Figure 2:**
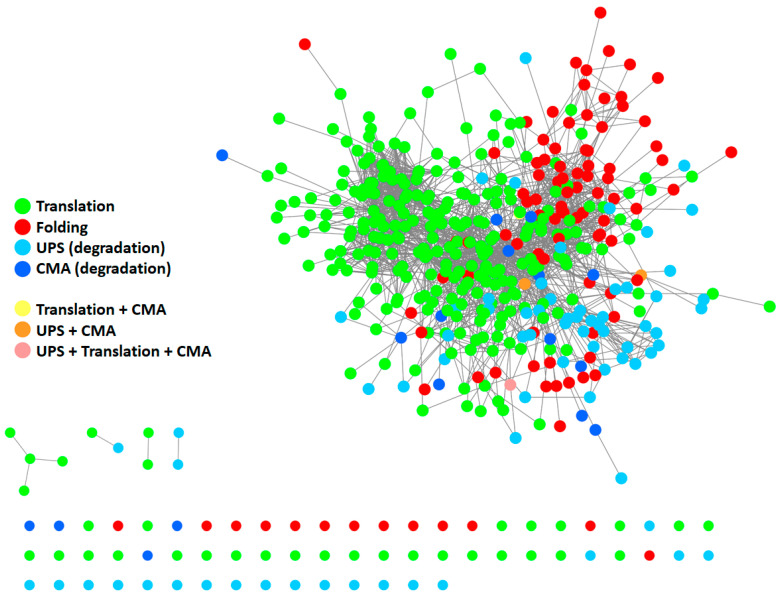
Protein–protein interaction (PPI) network of proteins participating in the PN. The network comprises 410 proteins. It was generated starting from four protein lists, encompassing all modules of the PN: protein translation (Reactome ID: R-HSA-72766.4; *n* = 294), protein folding (Reactome ID: R-HSA-391251.1; *n* = 102), ubiquitin-proteasome degradation (Reactome ID: R-HSA-8852135.2; *n* = 80), and chaperone-mediated autophagy (Reactome ID: R-HSA-9613829.3; *n* = 22). Information about interactions (edges) was retrieved in the IMEX database. Unconnected nodes are displayed at the bottom. UPS: ubiquitin-proteasome system. CMA: chaperone-mediated autophagy.

To give a representation of all proteins participating in the PN and their respective interactions, we generated a protein–protein interaction (PPI) network (Figure 2) using Cytoscape [18] and by retrieving information of interactions in the International Molecular Exchange Consortium (IMEx) database [19]. To this end, we merged four protein lists (Table S1) downloaded from the Reactome Pathway database [20], representing four pathways: “protein translation”, “protein folding”, “ubiquitin-proteasome degradation”, and “chaperone-mediated autophagy”. As shown in Figure 2, most proteins belonging to the four modules are highly interconnected with one another, thus forming a big network. Some proteins also participated in more than one pathway. This supports the idea that the PN modules cannot be considered as standalone modules and further underlines the highly interconnected nature of the PN.

### 2.1. Molecular Chaperones

The human genome encodes more than 300 chaperone molecules, which collectively constitute the “chaperome” [21].

Chaperones are roughly grouped based on their molecular weights and the majority of them are generally termed as “heat shock proteins” (HSPs). The main classes of chaperones in human cells are as follows: (i) HSP70s, (ii) HSP90s, (iii) HSP60s (or chaperonins), (iv) HSP100s, and (v) small ATP-independent HSPs (sHSPs). Their activity is assisted by additional co-chaperone molecules, namely the HSP40s as regulators of HSP70s and the tetratricopeptide repeat proteins (TPRs) as regulators of HSP90s.

The function and structure of both chaperones and their regulators have been extensively characterized and revised elsewhere [22,23,24]. Briefly, HSP70s assist protein folding by binding hydrophobic regions of client proteins in an ATP-dependent process. They constitutively have low ATPase activity and low substrate turnover rates, but this activity can be increased by the HSP40s co-chaperones, which also confer them substrate specificity. The HSP70 interacting protein (HIP) can also modulate the ATPase activity by delaying the release of ADP. HSP90s are homodimers which assist protein folding in endoplasmic reticulum, mitochondria, and cytoplasm. ATP hydrolysis mediates, in this case, the release of the client protein from the HSP90. HSP60s (usually termed chaperonins) contain a central cavity which shields the client protein from other proteins and prevents intra-molecule interactions. The opening/closing of the lid of the cavity is dependent on ATP binding and hydrolysis. HSP100s play a major role in resolubilizing and reactivating aggregated proteins. sHSPs prevent protein aggregation by binding to hydrophobic regions of misfolded proteins, but in an ATP-independent process.

One important aspect of chaperones function is that they interact to form modules and functional complexes, which work in cascades [25]. Indeed, specific chaperone pathways are involved depending on the protein clients. Some proteins can achieve their final folding after interaction with upstream chaperone molecules, such as HSP70s, while others would require more specialized downstream chaperones, such as HSP90s and/or chaperonins. In this frame, different chaperone systems cooperate as parts of a unique big network, and even a single malfunction in this network can alter the correct flux of protein folding.

### 2.2. Protein Degradation

Of the estimated 2000 components of the PN, the largest group (around 850 components) is represented by the ubiquitin–proteasome system (UPS), which is responsible for 80% of protein turnover in human cells [17,26]. The second main degradation machinery, the autophagy–lysosome pathway (ALP), also comprises around 500 components [27]. In addition to these two general mechanisms, specific proteases are involved in the fine tuning of the level of proteins which play highly regulated functions in cellular pathways (e.g., biosignalling, transcription, enzymatic cascades, cell cycle) [28].

Central to the UPS are the ubiquitylation of target proteins and the proteolysis within the proteasome complex. On the other hand, autophagosome formation and fusion with the lysosome are central to the ALP. Both processes are finely regulated within cells in order to maintain the correct protein homeostasis as a balance between synthesis and degradation. The two processes display a certain degree of specificity in terms of substrates. One of the main differences between the UPS and ALP is that the latter can directly remove insoluble protein aggregates and larger inclusions by the engulfment in the autophagosome. Nevertheless, the UPS and ALP are strictly interconnected, and it has been demonstrated that they can compensate one another when overwhelmed or dysfunctional [29,30]. This further highlights the network character of the PN.

## 3. The Failure of the PN in Neurodegeneration

Age represents the most important risk factor for the development of many neurodegenerative disorders. One of the reasons for this is the unavoidable decline in the ability of human cells to maintain the correct proteostasis. Why the PN deteriorates with aging is still not completely clear, however, the lack of evolutionary pressure for proteome maintenance beyond the reproductive age surely contributes.

Independently of the causative mechanisms, a hallmark of the aging proteome is decreased protein solubility, accompanied by the accumulation of aggregates. This process has been extensively studied in *Caenorhabditis elegans*, where it has been demonstrated that low abundant proteins display higher aggregation propensities than the highly abundant ones. However, even though highly abundant proteins have greater intrinsic solubility, they actually contribute the most to the total aggregates [31]. Reasonably, their solubility is not sufficient to protect them from aggregation when the PN is deregulated and proteins exceed their critical concentration within the cells.

PN deregulation and decline dramatically affect neurons. Indeed, misfolded and/or oxidized proteins mainly accumulate in non-dividing, long-lived cells [32]. Moreover, in the human brain, the expression of ATP-dependent chaperones is reduced with age, thus promoting misfolding and aggregation [21]. When the efficiency of the PN falls below a critical level, the aggregation-prone proteins cannot be maintained in a soluble state. This threshold level can also be lowered by additional stress conditions or in the presence of mutations that affect specific proteins, thus further promoting aggregation in a positive feedback loop.

Alzheimer’s disease (AD), Parkinson’s disease (PD), Huntington’s disease (HD), and amyotrophic lateral sclerosis (ALS) are examples of neurodegenerative proteinopathies [33,34]. During disease progression, the accumulation of toxic protein aggregates is favoured by PN malfunction, and the activity of PN components, in turn, is affected at several levels by the presence of the aggregates. This triggers a vicious cycle that leads to proteostasis disruption and eventually cell death.

Even though different pathogenetic mechanisms underlie the progression of different neurodegenerative proteinopathies, some shared features linked to PN failure can be identified. Indeed, chaperone molecules and degradation machineries can be sequestered into the pathological aggregates [35,36,37], the UPS is usually overwhelmed [38,39], and the general cellular ability to cope with stress conditions is hampered [40,41]. These mechanisms could be targeted in an attempt to ameliorate and to lower proteinopathies progression.

## 4. Targeting the PN in Neurodegeneration: “Classical” Pharmacological Approaches

Due to the role of PN in maintaining the correct proteostasis, the easiest pharmacological approach that can be explored in neurodegenerative proteinopathies is to boost the system. This can be achieved by either modulating the activity of individual components or acting broadly on the master regulators of the PN. These targeted pharmacological approaches proved to be effective in ameliorating some proteinopathies, as extensively revisedelsewhere [42]. Here, we discuss some of these strategies, with the aim of also highlighting the limitations of such approaches.

The upregulation of HSP40s represents a viable strategy, since mutations in some of these proteins have been linked to neurodegeneration [43]. Three HSP40 family members are of particular interest, namely DNAJB2, DNAJB6, and DNAJB9, which bind polyQ-containing proteins, α-synuclein, and β-amyloid, respectively. One small compound has been described which increases HSP40s activity, thus improving the function of the HSP70s system [44]. Also, the enhancement of HIP function, thus increasing HSP70s activity, seems to inhibit the aggregation of misfolded proteins [45,46].

By contrast, the inhibition of HSP90s has been proposed as a therapeutic strategy in neurodegeneration. Indeed, when the activity of HSP90s is reduced, HSP70s levels are increased in turn, thus enhancing the degradation of toxic protein aggregates (e.g., tau and polyQ-containing proteins) [47]. Moreover, both α-synuclein and tau are HSP90s client proteins, and it has been proposed that HSP90s contribute to the stabilization of the toxic intermediates [48]. However, the systemic inhibition of HSP90s is toxic and the use of brain penetrant inhibitors, originally developed as antineoplastic drugs, is also neurotoxic [49,50].

The activity of chaperonins can also be targeted as a therapeutic strategy in neurodegeneration. As an example, the mHtt protein, which forms toxic fibrils in Huntington’s disease, is a known client protein of TRiC, a cytoplasmic 1 MDa complex composed of members of the HSP60 family [51]. Also, in this case, an indirect approach to increase the levels of TRiC has been proposed, namely, the use of small molecules for the inhibition of the VRK2 kinase, which normally inhibits the activity of the USP25 deubiquitinase, which deubiquitinates TRiC. Thus, the inhibition of VRK2 results in increased levels of TRiC, which is no longer degraded via the UPS. It has been demonstrated that such inhibitors are effective in reducing the aggregation of mHtt [52].

Since a general upregulation of the chaperone system is expected to ameliorate neurodegeneration, some efforts have focused on the HSF1 protein, which is recognised as the master transcription factor that regulates the activity of the PN [53]. HSF1 is normally responsive to stress; thus, small molecules that induce HSF1 act through non-specific stressful mechanisms, which makes them of little use as therapeutics. A small molecule has been recently identified, namely HSF1A, which activates HSF1 without causing cellular stress and increases the levels of HSP70s, thus reducing protein aggregates in various mammalian cells and fruit fly models [54,55].

Even though molecular chaperones seem to be promising targets, the tuning of the other modules of the PN (i.e., protein synthesis and degradation) also represents a good strategy. For instance, the use of small molecules which boost the UPS and/or ALP system has been proposed [56,57,58] in order to improve protein clearance, thus ameliorating neuron cells viability. On the other hand, reducing the translational activity represents an equally promising strategy. Indeed, reducing the levels of newly synthesized proteins results in the unburdening of the downstream PN modules. A general reduction of protein translation is always observed in response to stress when the cellular energy needs to be saved to cope with the adverse conditions. In this frame, the pharmacological lengthening of stress-induced translational attenuation has been proved to be effective in proteinopathies [59,60], even though it cannot be envisioned as an actual therapeutic strategy.

## 5. Novel Strategies to Ameliorate Proteinopathies: The “Network Medicine” Approach

As already described, the PN comprises around 2000 components functionally grouped into three main modules. These modules are deeply interconnected, and all components work together and compensate one another to maintain proper protein homeostasis. The malfunction of even a single or a small group of components unavoidably impacts the whole PN, with consequences that are difficult to predict if every single component is considered as a standalone.

### 5.1. Complex Systems and Diseases

Biological organisms are complex systems made of individual components which interact at different levels, thus creating a complex network. In this frame, any possible alterations which impact the function of a single component (e.g., a specific gene mutation) will introduce a perturbation in the entire network. Typically, complex systems are capable of reacting to both internal and external changes by the reorganization of their individual components, which results in the acquisition of novel properties. These properties of the system can be explained only in the frame of the systems theory [61,62]. Indeed, an “emergent property” is a characteristic of the system that is not present in its individual components but arises from the collaboration among the elements of the system.

The concept of “emergence” is perfectly suitable to multifactorial diseases, in which multiple factors can be associated with the pathogenetic process. Neurodegenerative diseases belong to this category, since the diseased state is usually the result of multiple genetic and environmental causes. Even in familiar forms (e.g., PARK2-mutated PD patients), where a clear genetic alteration is recognized as the main etiological driver, the clinical phenotype can vary, based on the co-occurrence of additional factors. Moreover, complex diseases always display nonlinear correlation between genotype and phenotype, which means that the same genotype can result in different phenotypes and also that the same disease phenotype can arise from different genotypes. One example is the GGGGCC hexanucleotide repeat expansion in the C9orf72 gene, which is associated with both ALS and frontotemporal dementia (FTD) [63]. On the other hand, several gene mutations (i.e., the so-called PARK PD-associated loci) are associated with familiar PD as a clearly defined clinical phenotype [64].

Complex biological systems are efficiently represented by networks. Graphically, a network is made of nodes, which usually represent genes or proteins, connected to one another by edges, which represent relations between nodes (e.g., physical interaction, functional link, co-expression). Some nodes, called “hubs”, display a high number of connections, while others are located at the periphery and have a few connections. Networks can be classified as either random or scale-free [65]. In random networks, the connections among the nodes are, by definition, “random”, which means that each node has the same probability of being connected to any other node. By contrast, in scale-free networks, the probability distribution of the number of links per each node follows a power-law distribution: the network will include some (a few) highly connected nodes (i.e., hubs) and many nodes with few connections. Biological networks are scale-free; this minimizes the detrimental consequences of most biochemical/genetic errors (which more likely affect a few connected nodes), unless the target of perturbations are hubs. Indeed, it has been demonstrated that hubs match to “essential” genes, whose mutation often leads to embryonic lethality [66]. On the other hand, human disease genes, independently of the type of disease, usually match with highly connected nodes, but not with hubs. By analysis of the connections among all known disease genes, it emerged that genes involved in similar diseases are frequently connected to one another. From this evidence, a “human disease network” was generated [67], where nodes represent diseases and edges represent the existence of shared disease genes. The shape of this network supports the idea that disease-specific functional modules exist.

As an example in the context of neurodegenerative diseases, Lim and colleagues applied systems analysis to identify disease-modifying proteins that are in common in neurodegenerative diseases which cause ataxia [68]. They generated a PPI network and demonstrated that a panel of genes associated with inherited ataxia, seemingly unrelated to one another, actually shared some interactors, which could act as disease-modifying genes in animal models.

On these bases, it is clear that studying the function of a single (or a few) disease-gene(s)/protein(s) will never give a comprehensive view of the pathogenetic process. Furthermore, from a pharmacological point of view, targeting a single disease-associated protein might not represent an effective strategy or may even be misleading.

### 5.2. Network Medicine

The “network medicine” approach has been proposed to overcome the main limitations of the classical targeted approach in medicine [69]. This novel approach is founded on the observation that a disease is almost never the result of a single protein dysregulation but usually reflects alterations of a complex intracellular network. Thus, targeting a single component of a complex network based on its involvement in a disease can represent an effective therapeutic strategy, but also entails several limitations. First, the outcomes are not completely predictable. For instance, the inhibition/boosting of a chaperone molecule can trigger downstream events, such as compensation mechanisms from other chaperones. Second, there is never only one real target. Every single component of the PN displays at least a small group of direct interactors (either physical or functional), whose activity is obviously also affected. Targeting a single component that plays a central role in the network can disrupt the entire network. For instance, the inhibition of HSP90s, which are able to increase the levels of HSP70s, resulting in beneficial effects on protein aggregation, is actually not feasible due to the fact that HSP90s are central highly connected nodes (so-called “hubs”) in the PN.

The classical reductionist view of the “one-target” pharmacology usually fails to identify novel therapeutics for complex diseases. In this frame, “network pharmacology” represents a way to innovate drug discovery. It takes advantage of the networks generated from the integration of “omics” data through the tools of systems biology. This network-centric approach is able to (i) suggest novel targets, (ii) identify hidden pathways involved in the disease, (iii) predict the effects of a treatment considering the entire system, (iv) suggest the dosing of a drug, based, for instance, on metabolic profiling, and (v) identify the causes of drug resistance and/or toxicity based on the “shape” (e.g., robustness, connectivity, fragility) of the network.

**Figure 3:**
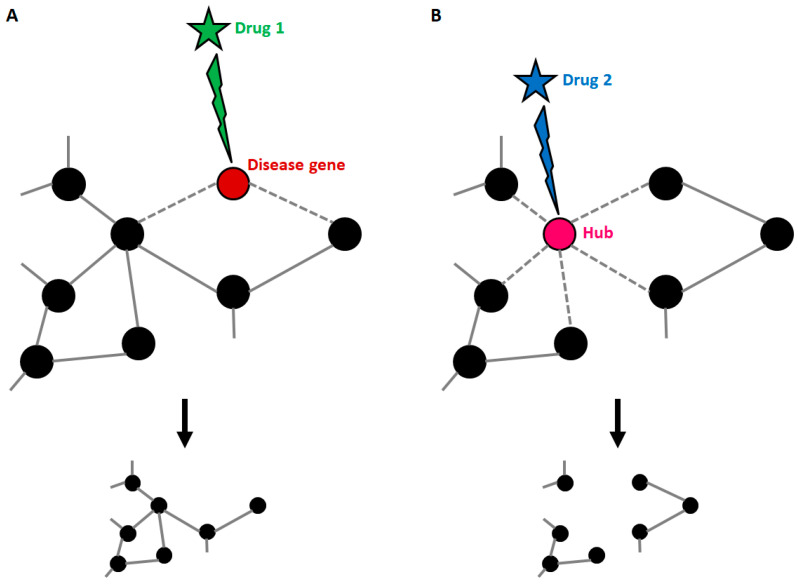
Druggable targets in the context of a disease network. (**A**) Disease genes usually represent nodes with an intermediate number of connections. If such genes are targeted by a drug, the treatment can be effective, with small impacts on the general structure of the network. (**B**) When a highly connected node (hub) is targeted by a drug, the entire structure of the network is affected with consequences that cannot be predicted if connections are not known. This explains why hubs usually match with the so-called “essential genes”.

As stated before, disease genes can harbour many connections, and this implies that targeting the products of known disease genes can dramatically impact on the whole network. Strikingly, it has been demonstrated that most approved drugs are actually “palliative” drugs; in other words, they do not target disease-associated proteins but proteins in their neighbourhood [70]. However, the degree of connectivity of disease genes within disease networks is still debated, since it has been demonstrated that some disease genes are restricted to the periphery of the interactome [69]. Whatever the location of disease genes, knowledge about the composition of the disease network is the only tool available to identify candidate disease-modifying products to be targeted by drugs (Figure 3). Yildirim and co-workers generated a “drug-target” network [70], in which FDA-approved drugs are linked with their target proteins. In this network the majority of drugs were highly connected to each other, thus forming a giant component. Notably, it also emerged that many “old” drugs targeted already targeted proteins; by contrast, new effective drugs used in combined therapeutic treatments showed a trend toward more functionally different targets. This clearly suggests that the failure of the classical “one target” pharmacological approach resides in the fact that new drugs were still designed to target already well-known proteins.

Network pharmacology has not yet been applied extensively to proteinopathies. Recently, in the frame of novel network-based approaches, the opportunity to use autophagy enhancement as a strategy against α-synuclein aggregation in PD has raised considerable interest [71]. It is well known that ALP is an important pathway in α-synuclein degradation. Since rapamycin and other early macroautophagy enhancers display limited selectivity, some novel molecules have been proposed which target this module of the PN in an indirect way, acting mainly at the lysosome level. Currently, ambroxol is in phase II clinical trials for the treatment of PD and PD with dementia as a repositioned drug, based on its ability to solubilize protein aggregates [72].

Less than one year ago, a network-based methodology appeared to be able to identify efficacious drug combinations for specific diseases [73]. The authors developed a strategy to quantify the relationship between disease proteins and drug targets in the human interactome network. They demonstrated that a drug combination is effective only when it follows a specific network topological relationship to the respective disease module. Specifically, to obtain a synergic effect, the two drug-target modules must overlap with the disease module but must also be separated (low network proximity) in the human interactome network. This tool represents a unique opportunity to develop novel drug-combination therapies for several complex diseases, including neurodegenerative proteinopathies.

## 6. Conclusions

Neurodegenerative proteinopathies represent a class of complex diseases whose pathogenetic mechanisms are still being thoroughly investigated. The failure of the PN clearly represents a main trigger in the onset and progression of such diseases. Even though several etiopathological mechanisms have been unveiled, and many of them represent common altered pathways in different diseases, disease-modifying treatments are still lacking, and patients suffering from these disorders can only rely on palliative drugs. In this frame, network medicine represents an opportunity to overcome the main limitations of the classical “one target/one drug” pharmacological approach. Network pharmacology will indeed help in the development of new strategies for rational drug design, taking into account that each cellular component cannot be considered as a standalone. Moreover, the combination of different omics analyses performed in the same patient will allow for the generation of patient-specific networks at different levels, which will guide clinical decisions in the frame of personalized medicine.

Some network-based approaches that are able to predict the effectiveness of some drug combinations based on the “quantitative” analysis of the drug–target network in the context of the protein interactome have already been developed. Still, they deserve to be implemented and generalized in order to become useful tools for all complex diseases.

## Supplementary Materials

The following are available online at https://www.mdpi.com/1422-0067/21/17/6405/s1, Table S1. Protein lists used to generate the PPI network of the PN.
